# Inorganic nanomaterials for intelligent photothermal antibacterial applications

**DOI:** 10.3389/fbioe.2022.1047598

**Published:** 2022-10-21

**Authors:** Bao Wang, Yan Xu, Donghan Shao, Leijiao Li, Yuqin Ma, Yunhui Li, Jianwei Zhu, Xincui Shi, Wenliang Li

**Affiliations:** ^1^ School of Chemistry and Environmental Engineering, Changchun University of Science and Technology, Changchun, China; ^2^ Zhongshan Institute of Changchun University of Science and Technology, Zhongshan, China; ^3^ Engineering Research Center of Antibody, Jilin Medical University, Jilin, China

**Keywords:** inorganic nanomaterials, photothermal therapy, near infrared-induced, antibacterial, biomedicine

## Abstract

Antibiotics are currently the main therapeutic agent for bacterial infections, but they have led to bacterial resistance, which has become a worldwide problem that needs to be addressed. The emergence of inorganic nanomaterials provides a new opportunity for the prevention and treatment of bacterial infection. With the continuous development of nanoscience, more and more inorganic nanomaterials have been used to treat bacterial infections. However, single inorganic nanoparticles (NPs) are often faced with problems such as large dosage, strong toxic and side effects, poor therapeutic effect and so on, so the combination of inorganic nano-materials and photothermal therapy (PTT) has become a promising treatment. PTT effectively avoids the problem of bacterial drug resistance, and can also reduce the dosage of inorganic nanomaterials to a certain extent, greatly improving the antibacterial effect. In this paper, we summarize several common synthesis methods of inorganic nanomaterials, and discuss the advantages and disadvantages of several typical inorganic nanomaterials which can be used in photothermal treatment of bacterial infection, such as precious metal-based nanomaterials, metal-based nanomaterials and carbon-based nanomaterials. In addition, we also analyze the future development trend of the remaining problems. We hope that these discussions will be helpful to the future research of near-infrared (NIR) photothermal conversion inorganic nanomaterials.

## 1 Introduction

The risk of bacterial infection is common in living environment and public facilities. Especially after surgery, the risk of infection with pathogenic bacteria is extremely high and difficult to treat (Sethulekshmi et al., 2022). Minor delay wound healing, and heavy produce life-threatening complications. Bacterial infections have become one of the enemies of human health. In order to effectively fight against bacterial infections, a series of antibiotics are manufactured and widely used, such as: penicillin, roxithromycin, vancomycin, cephalosporin, etc. (Hochvaldová et al., 2022). But antibiotic abuse has led to bacterial resistance, forming multiple multidrug-resistant (MDR) bacteria, such as methicillin-resistant *Staphylococcus aureus* (MRSA), vancomycin-resistant *Staphylococcus aureus* (VRSA) and vancomycin-resistant Enterococcus (Abbas et al., 2022). It is reported that 10 million people may die every year from 2022 to 2050 due to bacterial antibiotic resistance (O'Neill 2016). Antimicrobial-resistant (AMR) and multidrug-resistant (MDR) infections will have a higher mortality rate than all types of cancer combined, according to projections from the Centers for Disease Control and Prevention (CDC) (Pormohammad et al., 2021). Therefore, it is of great significance to develop unconventional antibiotic drugs to treat bacterial infections (Chaudhary 2016).

In recent years, nanotechnology has become increasingly linked to biomedicine, and some nanomaterials with antibacterial activity are considered an ideal way to treat and prevent bacterial infections. Nanomaterials generally refer to materials in the range of 1–100 nm in at least one dimension in three-dimensional space. Inorganic nanomaterials are widely used in the treatment of tumors, cancers and bacterial infections due to their low toxicity, small size, good biocompatibility, easy modification and large surface energy (Chaudhary et al., 2016). At present, a variety of inorganic nanomaterials have been synthesized, including: metal-based nanomaterials, transition metal-based nanomaterials and carbon-based nanomaterials. The size and morphology of nanomaterials are the main indicators affecting their performance, and among the nanomaterials that have been synthesized, the morphology mainly includes: nanorods (Zhang et al., 2021), nanospheres (Li et al., 2022), nanowires (Han et al., 2022), nanoflower (Mutalik et al., 2021), nanotubes (Zhao et al., 2023), nanosheets (Mutalik et al., 2022), etc. The unique antibacterial mechanisms of nanomaterials are the following: 1) Destruction of bacterial DNA and proteins through the release of metal ions or the production of reactive oxygen species (ROS); 2) Destruction of bacterial cell membranes through the aggregation of nanoparticles (Qi et al., 2020); 3) NPs accumulate on the surface of the cell membrane to block the transmembrane transmission of electrons; 4) Heat shock/stress caused by temperature increase (Hamblin 2016; Wang C. et al., 2020). However, many times a single nanomaterial has problems such as insignificant therapeutic effect, large dose of material used, and strong toxicity in the treatment of bacterial infection. To this end, the search for safer and more effective treatments for bacterial infections has become a major focus for scientists.

As a controllable and non-invasive treatment method, PTT has been widely used in biomedical fields as a new treatment model in recent years. The PTT converts light energy into thermal energy through physical means, and a photothermal agent is required during the conversion process. It has become a popular trend to carry out nanomaterials as photothermal agents in combination with photothermal therapy and applied in antibacterial therapy. The advantages of photothermal therapy are mainly in the good penetration of tissues, and this non-contact treatment can effectively avoid bacteria developing drug resistance. Ultraviolet light (<380 nm), visible light (380–760 nm), and NIR light (>760 nm) are commonly used light sources for several photothermal therapies. The wavelength of visible light is between 380 and 760 nm, accounting for about 52% of the solar spectrum, and the wavelength of ultraviolet light is less than 380 nm, which is in the invisible region along with NIR light, accounting for 4% and 44% of the solar spectrum, respectively. By adjusting the band structure engineering of the photothermal agent, the surface plasmon resonance effect or the addition of light enhancers to reduce the very small proportion of ultraviolet light in the solar spectrum, resulting in its photothermal conversion efficiency cannot be satisfactory. Therefore, achieve full use of the light source is a very meaningful thing (Wang L. et al., 2021).

**FIGURE 1:**
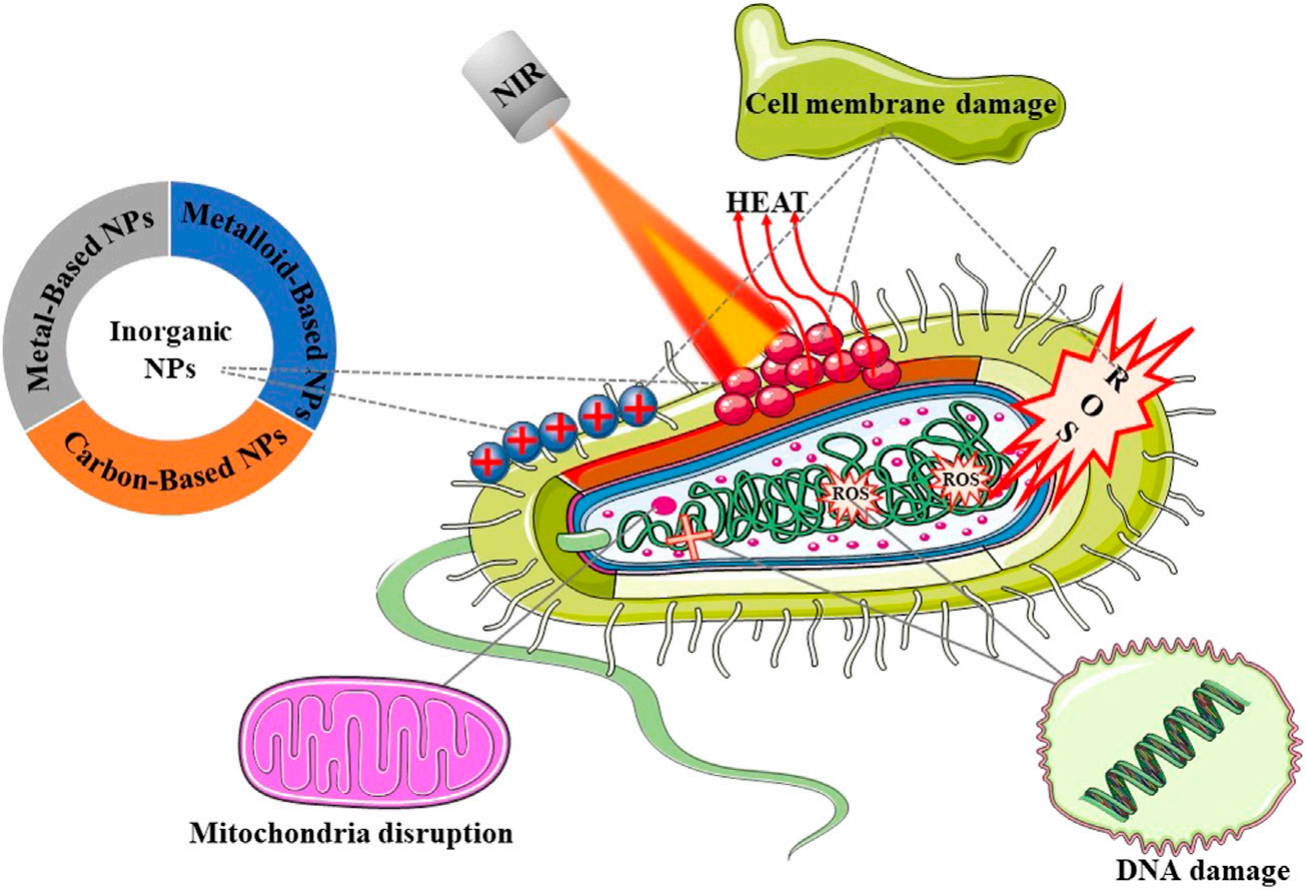
Schematic illustration of the main topics covered in this review.

Inorganic nano-photothermal conversion materials for the treatment of bacterial infections are fully discussed here (Figure 1). Starting from the synthesis method, the preparation and modification methods of inorganic nanomaterials of different morphologies and sizes, the antibacterial mechanism under different light sources, and various factors affecting the photothermal conversion efficiency of nanomaterials are described. Finally, the application of photothermal conversion nanomaterials in antimicrobials is described, and its future development direction and challenges are prospected. Here, we summarize several synthesis methods of inorganic nanomaterials and their advantages and disadvantages (Table 1), as well as the advantages and antibacterial mechanism (Table 2) of several inorganic nanomaterials used in photothermal treatment of bacterial infection.

**TABLE 1:** Synthesis methods and advantages and disadvantages of Inorganic nanoparticles.

Synthesis method	Synthesis principle	Advantages	Disadvantages	References
Radiation induced	Irradiate a solution containing precursors with accelerated electrons or gamma rays	Not affected by temperature, and the purity of the product is high	Radiation does great harm to human health and has the risk of causing cancer	Güven (2021)
Biosynthesis	Extract required elements from plants or organisms	The raw materials are cheap and easy to obtain, and the biosafety is high	The extraction process is complicated and the output is low	Rambabu et al. (2021)
Seed mediated method	The extremely small nanocrystals were added to the supersaturated solution for secondary nucleation	Avoid the homogeneous nucleation process which is difficult to control	The purity of the reaction reagent is high, and the concentration of the precursor is not easy to be controlled	Shi et al. (2021)
Assisted synthesis of capping agent	The capping agent is selectively adsorbed on the surface of nanocrystals by changing the surface free energy	Nanoparticles with multiple morphologies can be obtained by using end-capping agents	The dispersion and solubility of nanoparticles will be affected	Huang et al. (2020)

**TABLE 2:** Summary of inorganic nanomaterials for NIR photothermal antibacterial.

Nanoparticles	Advantage	Treatment strategy	References
Au NPs	It has strong light absorption, surface plasmon resonance and photothermal effect	When used in photothermal therapy, the lattice is heated, the temperature of the material rises, and bacteria are killed	Ma et al. (2019)
Ag NPs	It has adjustable surface plasmon resonance effect, has certain antibacterial properties and is easy to synthesize	Ag itself can interact with phosphate and other substances inside and outside the cell, and inhibit the growth of bacteria. When combined with NIR, it can destroy the cell membrane and lead to bacterial death due to local heating	Tamboli and Lee (2013)
B NPs	It has the functions of anti-inflammation and metabolic regulation, can maintain the stability of cell membrane and has targeting	The existence of element B promotes the separation of holes and electrons, improves the optical properties, and can produce more ROS to kill bacteria under light conditions	Ran et al. (2022)
Te NPs	It can specifically bind to glutathione in cells and has a certain antibacterial effect	Te (0) accumulates in cells, produces ROS under NIR laser irradiation and activates intracellular oxidative stress, resulting in the death of bacteria	Huang et al. (2022)
CQDs	It has small size, good water dispersibility, biocompatibility and optical properties, and is easy to be removed from the body	CQDs can produce ROS under laser irradiation, and the temperature increases at the same time	Ghirardello et al. (2021)
Graphene	It has good thermal conductivity and optical transmittance and has full spectrum absorption	Graphene itself has a targeted effect on bacteria, graphene produces high temperature under NIR laser irradiation, and the two synergistically enhance the antibacterial effect	Wang H. et al. (2020)

## 2 Synthesis of inorganic nanomaterials

The synthesis of nanomaterials is usually mainly divided into “top-down” and “bottom-up” methods (Saravanan et al., 2021). Top-down generally refers to the decomposition of large pieces of material into smaller parts by physical or chemical methods such as ultrasonic crushing or laser etching (Nekoueian et al., 2019; Cui et al., 2022). Bottom-up generally refers to the formation of new materials by assembling different materials together by chemical or biological means such as one-pot method or biosynthesis (Jamkhande et al., 2019). In view of the fact that the internal stresses introduced by top-down synthesis methods in many cases will destroy the morphology of nanomaterials and affect the surface effect of NPs, most nanomaterials choose bottom-up methods for synthesis (Xia et al., 2019). Commonly used bottom-up synthesis methods are mainly as follows:

### 2.1 Radiation induced

Radiation induced technology has become a very popular method for the synthesis of nanomaterials because it is simple, fast, green and is not affected by the experimental temperature. It has been widely used in the fields of medicine and environment (Xiong et al., 2021). In the process of synthesis, the solution containing the corresponding precursor can be irradiated with accelerating electrons or gamma rays to synthesize NPs with smaller size (Čubová and Čuba. 2019), and high-purity products can be obtained without strictly controlling the experimental conditions. Ionizing radiation with high-energy gamma photons and accelerated electrons can uniformly generate free radicals in the transmitted medium (Güven 2021).This property makes ionizing radiation technology widely used in the preparation of nanomaterials.

**FIGURE 2:**
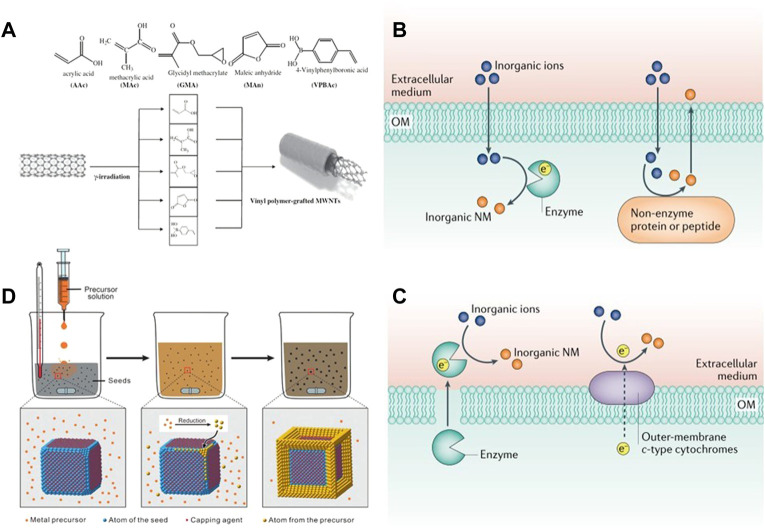
**(A)** Linking the surface of MWCNT’s with different vinyl monomers by laser irradiation. Reproduced from (Yang et al., 2010) with permission from Radiation Physics and Chemistry. The biosynthesis of inorganic nanomaterials can be generated by **(B)** secreted enzymes and membrane proteins extracellularly, or **(C)** intracellularly, by combining proteins/peptides with inorganic ions or oxidoreductases. Reproduced from (Choi and Lee. 2020) with permission from Nature Reviews Chemistry. **(D)** General procedure for obtaining desired nanocrystals from metal precursors using a seed-mediated method. Inorganic-nanomaterial synthesis in microbial cells and bacteriophages. Reproduced from (Xia et al., 2016) with permission from Angewandte Chemie.

By irradiating the carbon nanotubes with *γ* ray, the carbon nanotubes can be uniformly dispersed and kept stable without using a dispersant (Saif et al., 2018) (Figure 2A). Radiation induced technology is also commonly used in the synthesis of quantum dots (Gidwani et al., 2021). Li et al. (2011) Synthesized SnSe quantum dots by using ionizing radiation technology and adding surfactant CTAB at room temperature, which effectively avoided the problems of high synthesis temperature and the use of toxic precursors. In addition to the above nanoparticles, nano gel can also be synthesized by radiation-induced method. Recently, Sabatino et al. (2020) reviewed the progress made by *γ* ray, electron beam and ultraviolet radiation are used to mediate the formation of nano gel, and the application of the synthesized gel is briefly summarized (Matusiak et al., 2020; Sabatino et al., 2020). Radiation induced technology provides a simpler way for the synthesis of inorganic nanoparticles and is expected to be widely used in the future synthesis process.

### 2.2 Biosynthesis

Green synthesis is one of the effective methods to reduce the biological toxicity of nanoparticles, which can effectively improve the biocompatibility of nanoparticles (Zonaro et al., 2015). In the process of green synthesis, biosynthesis mainly extracts the required elements from organisms or plant organisms. It is favored for its advantages of economy, safety and easy availability (Rambabu et al., 2021). There is no need to add additional reductant or capping agent during the synthesis process, nor high pressure and high temperature (Salem and Fouda 2021). Plants are more favorable for the synthesis of nanoparticles due to their various biological molecules such as polyphenols, proteins, flavonoids and bioactive amines (Lee et al., 2020). The method of synthesizing nanoparticles with plant extracts is a one-step synthesis method (Wang D. et al., 2019). Plant synthesis has the advantages of cheap and easy access to raw materials, low biological toxicity, high economic efficiency and safety. The synthesis of nanoparticles from plant extracts was first proposed by Gardea-Torresdey et al. (2003) using alfalfa sprouts to synthesize Ag-NPs. After that, some people successively proposed the biosynthesis of silver and gold NPs by golden rutin flower; Ag NPs with very small size (2–4 nm) were synthesized with Camellia sinensis (Rolim et al., 2019); Fe NPs with a size in the range of 30–100 nm were synthesized with tea leaves extract (Lin et al., 2020).

Microbial synthesis is the second largest biological source of nanoparticles. The mechanism for the synthesis of nanoparticles is that numerous metal or non-metal ions (such as Cu^2+^, Mg^2+^, Ag^+^, Cd^2+^) will interact electrostatically with the negatively charged cell membrane and adsorb to the surface of the cell membrane, transferring through the microbial membrane to the cell, which in turn activates the cell’s over-defence mechanism and is exported as a monomer by the cell using the efflux pump. The output monomer is the synthesized nanoparticles (Choi and Lee. 2020). The biosynthetic pathway of NPs can be divided into intracellular synthesis and extracellular synthesis (Figures 2B,C). The reducibility of enzymes is a very important factor for biomolecules to reduce metals and nonmetals. In extracellular synthesis, inorganic ions are mainly reduced to NPs by enzymes secreted by cells. For example, with Shewanella oneidensis MR-1, Pd NPs with a size of 10–100 nm can be synthesized in and out of the cell by enzymes secreted by the cell or enzymes on the extracellular membrane under the action of bioelectrochemistry (Wang W. et al., 2019). During intracellular synthesis, it mainly depends on reductase, polypeptide or non-enzymatic protein. Through the gene expression of protein MT, glutathione can be oligomerized into polypeptide PC to participate in the reduction of Cu^2+^, Ag^+^ and other inorganic ions (Jung et al., 2018), so that nanoparticles can be synthesized and grown in cells. For example, Ag nanospheres with a size of 20–70 nm can be synthesized by using the covering protein of *Pseudomonas* sp. and a wide range of secondary secretions (John et al., 2020). In addition, Fungi and yeast, also one of the bacterial genera, are also broadly used for the synthesis of NPs. The fungus itself has a large amount of mycelium, which enables it to synthesize nanoparticles with high efficiency (Kitching et al., 2022).

Biomolecules contain functional groups. In most cases, they can be used not only for the synthesis of nanoparticles, but also for the stabilization and functionalization of NPs (Choi and Lee 2020). This also provides a new idea for the application of nanoparticles in the field of antibacterial.

### 2.3 Seed mediated method

In the traditional sense, most nanocrystals are directly generated by using the one pot method in the presence of salt precursor and reducing agent, and by external conditions such as microwave, ultrasonic wave and current. Sometimes, the above methods cannot obtain ideal size and morphology, so Chen et al. (2013) proposed the seed mediated method to accurately control the synthesis of nanocrystals. The seed-mediated method divides growth and nucleation into two steps. Seed crystals of very small size (about a few nanometres) are added to a solution below supersaturated concentration to continue growth. It avoids the difficult to control homogeneous nucleation process by directing the newly formed atoms to nucleate twice on the surface of the pre-synthesised seeds (Xia et al., 2016) (Figure 2D).

The following problems should be paid attention to in the seed mediated method: 1) High purity reaction reagents should be used; 2) Control the concentration of precursor to ensure that no other reaction occurs except heterogeneous nucleation; 3) Be careful that the seed crystal is oxidized before use (Thanh et al., 2014). In order to ensure the accurate synthesis of crystal seeds, inert gas or capping agent is often introduced in the process of synthesis to protect the crystal seeds. The most typical seed mediated method is to use surfactants to prepare Au nanorods (GNRs). For example, cetyltrimethylammonium bromide (CTAB) is often used as a “soft template” to guide the directional growth of nanocrystals. This template is a simple and effective method to prepare rare and precious metal materials. Firstly, chloroauric acid was reduced by sodium borohydride in CTAB aqueous solution to prepare smaller Au seed solution. Secondly, the growth solution was prepared by reducing Au (III) complex ion to Au (I) complex ion with ascorbic acid (AA) in CTAB aqueous solution. Finally, a certain quantity of seed solution was added to the growth solution. The biggest advantage of seed mediated method is that any existing nanocrystalline seed can be added to the growth solution composed of capping agent or reducing agent to obtain high yield and new morphology nanocrystals (Shi et al., 2021).

### 2.4 Assisted synthesis of capping agent

The growth of nanocrystals usually requires stable control of capping agents (Loza et al., 2020). The addition of capping agent will have an important impact on the internal structure, surface structure and shape of nanocrystals, so it is widely used in nanomaterials and synthesis (Yang et al., 2019). At present, a great many capping agents have been reported to be used, such as biomolecules, anionic/cationic species, small molecules and macromolecular polymers (Abadi et al., 2022). The capping agent is mainly used to selectively adsorb on a plane of nanocrystals by changing the free energy of the surface to prevent further deposition and growth of atoms on the plane. There are many examples where the use of capping agent has changed the morphology of nanomaterials. For example, when fructose is used as capping agent to synthesize tellurium nanocrystals, tellurium nanorods are synthesized without fructose, and triangular tellurium nanostar is synthesized after fructose is added (Huang et al., 2020). In addition, when eight different chain lengths of CTAB (C_n_TAB, *n* = 2–16, Even) are used to synthesise GNRs, GNRs of different sizes from nanometres to micrometres can be obtained (Ye et al., 2013; Inaba et al., 2019).

A single capping agent cannot fully satisfy the basic needs of NPs in the synthesis process. In addition to morphology and size, dispersion and solubility are also important indicators to evaluate NPs. The future development direction will be toward the simultaneous use of two different types of capping agents, which can not only effectively improve the yield, but also obtain more nanocrystals with uniform size and good dispersion (Jana 2005; Zheng et al., 2021).

## 3 Antibacterial application and antibacterial mechanism of photothermal conversion inorganic nanomaterials

In clinical applications, there are high requirements for the biocompatibility of photothermal therapy nanomaterials. Although many nanomaterials reported so far have excellent photothermal conversion properties, their long-term safety is doubtful and it is difficult to be directly applied to clinical treatment. For example, metal NPs have poor biological metabolic capacity, and carbon-based materials have been shown to trigger many toxic reactions, such as oxidative stress and lung inflammation. Therefore, improving the biocompatibility of photothermal materials to provide better biodegradability and low toxicity is also a key factor.

In the treatment of bacterial infection, PTT has been widely used because it can effectively prevent bacteria from producing drug resistance. In view of the poor penetration of visible light to tissues, NIR light is often used as the light source of PTT. The commonly used NIR photothermal conversion nanomaterials mainly include noble metal-based nanomaterials, transition metal-based nanomaterials and carbon-based nanomaterials.

### 3.1 Noble metal-based nanomaterials

#### 3.1.1 Au

Noble metal-based nanomaterials have attracted extensive attention in photothermal therapy and imaging due to their surface plasmon enhanced fluorescence, plasma resonance effect and plasma photothermal effect. Among many noble metal nanomaterials, the preparation methods of nano Au have been very mature. Au nanoparticles have the benefits of easy surface modification and great biocompatibility. When irradiated with NIR light of a certain frequency, the Au NPs not only have high absorption capacity for light, but also can increase the absorption of light by the Au NPs through its adjustable regional plasmon resonance (LSPR). In view of the wonderful light absorption properties of Au NPs, they are often used as photothermal agents for the treatment of bacterial infections.

**FIGURE 3:**
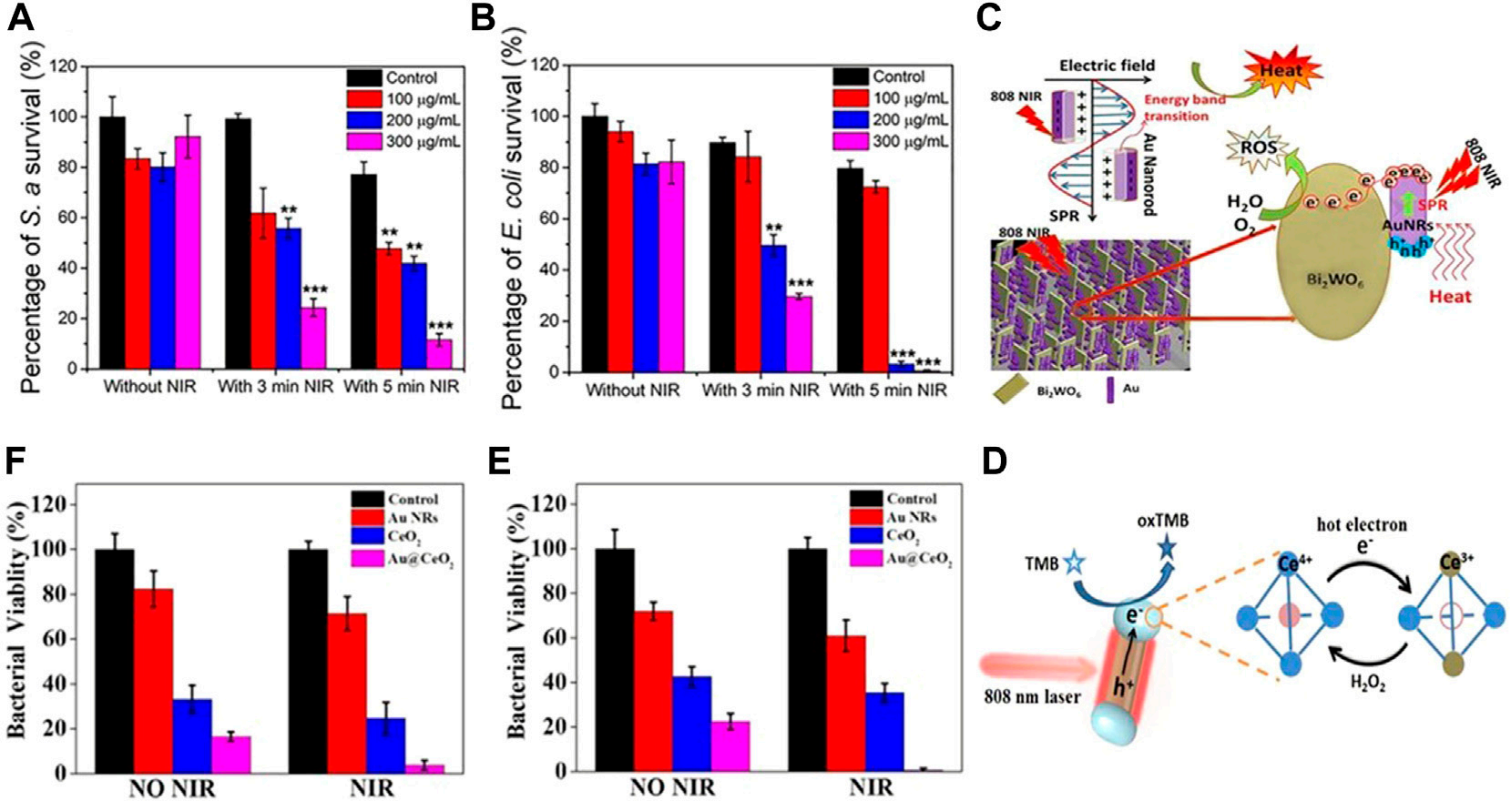
**(A)** Survival rate of *S. aureus* in corresponding group (*n* = 3); **(B)** Survival rate of E. *coli* in corresponding group (*n* = 3). Reproduced from (Ma et al., 2019) with permission from ACS Appl. Mater. Interfaces. **(C)** Photocatalytic mechanism of the Au@Bi2WO6 composite. Reproduced from (Li et al., 2019) with permission from Journal of Hazardous Materials. **(D)** Schematic diagram of carrier dynamics and carrier reaction of Au@CeO_2_ for peroxidase-like reaction under plasmon excitation. Enhanced antibacterial activity of Au@CeO_2_ nanozyme. Bacteriostatic activity of **(E)** *S. aureus* and **(F)** *E. coli* with different samples without and with NIR irradiation, respectively. Reproduced from (Liu et al., 2021) with permission from Applied Catalysis B: Environmental.

At present, the low photothermal conversion efficiency of Au is an urgent problem to be solved. In order to remove the application limitation brought by the photothermal conversion efficiency, scientists have explored the Au NPs with different morphologies and modified by different methods. GNRs are one of the most common morphologies in the synthesized gold nanomaterials, with strong absorption in the range of 600–900 nm (Vankayala and Hwang. 2018). When used for PTT, plasma resonance begins to attenuate with the heating of the lattice, resulting in an increase in the ambient temperature. In the process of photothermal conversion, the more electrons, the higher the thermal energy conversion. Based on this principle, Ma et al. (2019) constructed a core-shell nanostructure GNR@LDH of GNRs and layered double hydroxide (LDH). Under 2 Wcm^−2^ 808 nm laser irradiation, the temperature of individual GNRs could be increased by 31.8°C. GNR@LDH containing the same concentration of GNRs is 50°C warmer and can achieve a photothermal conversion efficiency of 60%.When using PEG GNR@LDH after functionalization, 300 μg ml^−1^ GNR@LDH-PEG (including 30 μg ml^−1^ GNRs) for 5 min, the bactericidal rates of *Escherichia coli* (*E. coli*) and *Staphylococcus aureus* (*S. aureus*) were 99.25% and 88.44%, respectively (Figures 3A,B). Compared with single component GNRs, noble metal semiconductor heterostructures exhibit new functions and better performance due to the synergistic effect between different components of heterostructures. The charge separation and light absorption at the heterostructure interface can be promoted by the surface plasmon resonance (SPR) of noble metals, which can effectively improve the photothermal conversion efficiency of GNRs (Leng et al., 2018). Li et al. (2019) combined GNRs and Bi_2_WO_6_ nanosheets, reduced the recombination of electrons and holes through the SPR effect induced by 808 nm NIR, improved the photocatalytic performance of Au@Bi_2_WO_6_ coatings, and was more likely to produce a large amount of ROS. When irradiated with NIR laser for 15 min, it was able to kill 99.96% and 99.62% of *E. coli* and *S. aureus*, and the sterilization rate was much higher than that when GNRs were used alone (Figure 3C). Sibidou et al. confirmed through experiments that the Au nanomaterials with biconical shape with (111) crystal plane have better photothermal effect than GNRs with (200) crystal plane (Yougbare et al., 2021a), and can kill all *E. coli* when irradiated at 808 nm for 7 min. In addition to the large Au nanostructures, the ultra-small gold nanoclusters also have ideal antibacterial effects. The use of biomolecules plays an important role in the synthesis of gold nanoclusters. Common biomolecules such as glucose, DNA, polypeptide, protein, etc. are reductive and have rich functional groups. They can not only act as reductants in the synthesis of gold nanoclusters, but also as ligands to realize the functionalization of Au nanoclusters (Pranantyo et al., 2019). Among many biomolecules, antimicrobial peptides (AMPs), as a kind of non-antibiotic biomolecules, can not only kill bacteria, but also be used as reducing agents to prepare gold nanoclusters with fluorescence emission. When the photothermal conversion efficiency reached 34.2%, the AMPs modified Au nanoclusters achieved the progressive killing of *E. coli* and HT-29 (Zhu et al., 2020) (Figure 3D). The unique SPR effect of noble metals enables it to be used in combination with a variety of materials to improve the therapeutic effect. GNRs can show a huge electric field enhancement at both ends of the rod under resonance excitation (Chen et al., 2013). Among them, the combination with nano enzymes has been proven to be effective in the treatment of bacterial infections. Liu et al. (2021) Deposited CeO_2_ nano enzyme at the end of GNRs to form a spatially separated dumbbell shaped nanostructure, which can simultaneously have plasma induced hot carriers and thermal effects (Figure 3E). Under 808 nm irradiation, Au@CeO_2_ The significant enhancement of peroxidase like activity of the Au composite enables the Au composite to have a broad-spectrum antibacterial effect (Liu et al., 2021) (Figure 3F).

The above antibacterial mechanism of Au NPs can be simply summarized as follows: Metal NPs are oxidized by NIR laser and released in the form of particles, generating ROS, destroying bacterial cell membranes, causing internal ribosomal instability, mitochondria and protein damage, and finally causing bacterial death (Faisal et al., 2013; Fu et al., 2014).

#### 3.1.2 Ag

Ag NPs have similar chemical properties to Au NPs. For example, large specific surface area, enhanced optical performance and the same LSPR effect. However, due to the size, shape, dosage and other reasons, Ag has non negligible biological toxicity.

**FIGURE 4:**
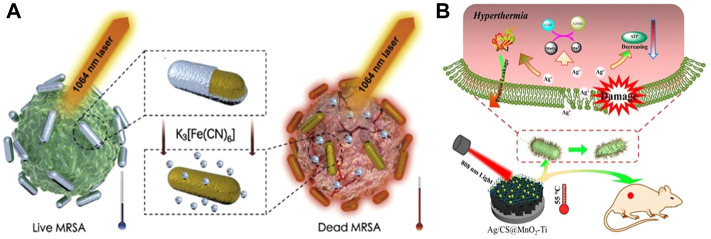
**(A)** Schematic diagram of NIR II combined with released Ag^+^ in the treatment of MRSA. Reproduced from (Mei et al., 2020) with permission from Biomaterials. **(B)** Synergistic antibacterial mechanism of Ag/CS@MnO_2_-Ti and 808 nm NIR. Reproduced from (Wang X. et al., 2019) with permission from ACS Appl. Mater. Interfaces.

Behravan et al. proved that the use of biosynthesis to obtain Ag NPs that can be used for the treatment of bacterial infection can effectively reduce the biological toxicity of Ag NPs, and the aqueous extract of Berberis leaves and roots was successfully used to synthesize Ag NPs with a size of 30–70 nm (Behravan et al., 2019). The antimicrobial performance was tested by disk diffusion method, which proved that it has a great inhibitory effect on *E. coli* and *S. aureus*. As a common biomolecule, polysaccharides also have low biological toxicity and are often used in the synthesis of hydrogels. Liu et al. (2020) embedded the Ag NPs synthesized with gallic acid (GA) into the network structure synthesized by polysaccharide carrageenan, and obtained a GA-Ag NP hydrogel with coagulation, air permeability, anti-dehydration and good bactericidal effect under 808 nm laser irradiation. It can also promote wound healing while treating bacterial infection. In the follow-up study, Mei et al. (2020) combined Ag and Au and also synthesized micro-Au/Ag NRs that can promote wound healing and can be used for the treatment of MRSA infection. On this basis, the micro-NPs can also be used for photoacoustic imaging (PA). *In vitro* and *in vivo* experiments showed that the synergistic effect of free Ag^+^ and 1,640 nm NIR could effectively eliminate MRSA and the biological toxicity was extremely low (Figure 4A). Meanwhile, activatable NIR PA can monitor the treatment process and provide timely feedback.

It has been proved in medicine that Ag nanoparticles have excellent antibacterial properties, and its antibacterial mechanism can be summarized as follows: when Ag NPs are used alone for antibacterial, Ag NPs have a high tendency to sulfur and phosphorus in cells, and can interact with phosphate and sulfur-containing proteins inside and outside cells. When the nanoparticles reach a certain concentration, they can achieve complete inhibition of bacterial growth (Tamboli and Lee, 2013). When Ag NPs are combined with PTT and hydrogel, under the irradiation of NIR light and heat, the hydrogel coated on the wound surface is locally heated, causing protein denaturation and membrane damage in bacterial cells (Liu et al., 2020), and promoting the release of Ag NPs. Ag NPs with larger surface area and smaller size are more likely to enter the cell and interact with thiol groups, causing bacterial death (Wang X. et al., 2019) (Figure 4B). Different antibacterial mechanisms show the great potential of Ag nano photothermal conversion materials in the biomedical field, and the combined treatment system of Ag NPs will effectively improve the therapeutic effect.

### 3.2 Metal like nanomaterials

#### 3.2.1 Boron

Boron (B) is one of the necessary trace elements for organisms. It can not only fight inflammation and regulate metabolism, but also maintain the stability of cell membrane (Xie et al., 2020; Duo et al., 2021; Jakubczak et al., 2021). In the past decade, many boron containing drugs have been approved for clinical use. However, due to potential toxicity, some boron compounds are still in the research stage. In recent years, researchers have paid much attention to the property that boron compounds can transform between neutral triangular plane sp^2^ and tetrahedral hybrid state sp^3^.

**FIGURE 5:**
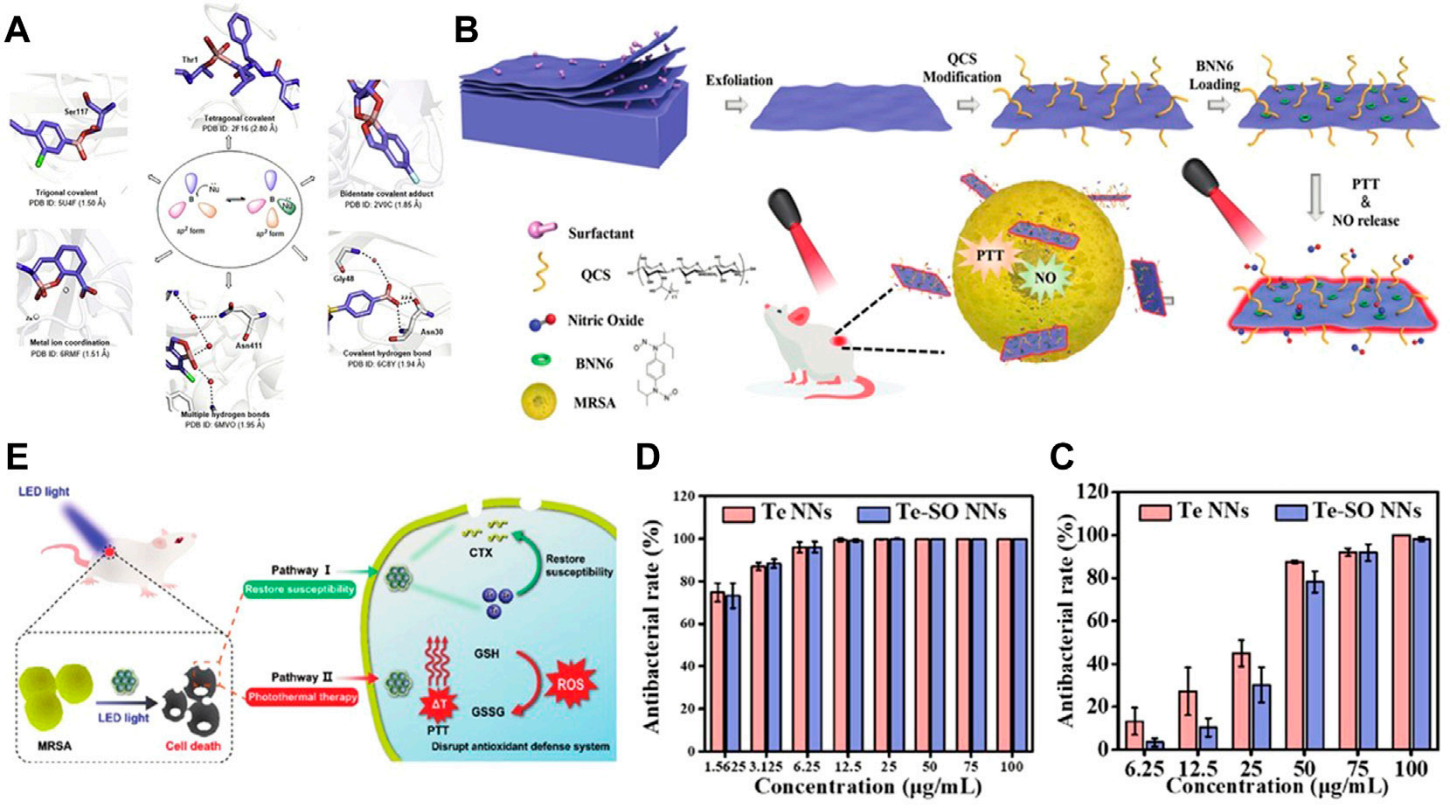
**(A)** Boron compounds and their interaction modes with biological targets. Reproduced from (Song et al., 2021) with permission from Acta Pharmaceutica Sinica B. **(B)** Preparation of B-QCS-BNN6 and its application in photothermal-NO synergistic antibacterial. Reproduced from (Lv et al., 2022) with permission from Biomaterials Science. Antibacterial activity of Te and Te-SO nanoneedles (NNs) toward **(C)** *S. aureus*. **(D)** and *E. coli*. antibacterial rate in the presence of Te and Te-SO NNs at different concentrations. Reproduced from (Huang et al., 2022) with permission from Materials Today Bio. **(E)** Bactericidal action of Te–CTX nanocomposites. The intracellular CTX and PTT synergism against MRSA for accelerating wound healing in mice. Reproduced from (Wu et al., 2022) with permission from Journal of Materials Chemistry B.

Boron derived compounds play a significant antibacterial role. In many previous studies, boric acid was considered as a promising drug that can resist antibiotic resistance (Song et al., 2021) (Figure 5A). Since 2015, tuberculosis (TB) caused by Mtb has caused about 250,000 deaths every year, and the treatment success rate of multi drug resistant tuberculosis (MDR-TB) is only 56%. It is reported that boric acid can treat tuberculosis by selectively killing MTB, and can also be used as an inhibitor of *Staphylococcus aureus caseinolytic* protease P (SaClpP) (Song et al., 2021). Under the driving of visible light, the boron doped TiO_2_ thin film can exhibit the inhibitory effect on *S. aureus*, *Acinetobacter* baumannii and *Streptococcus* pyogenes through the combined action of photocatalysis and B (Wong et al., 2020; Wang et al., 2022). The doping of B increases the specific surface area of nanoparticles, promotes the separation of electrons and holes, further improves the photocatalytic activity, and can produce a large number of ROS under light conditions, thus greatly improving the antibacterial activity (Ran et al., 2022).

Recently, someone proposed a photothermal antibacterial mode for B nanosheets. The multifunctional nano platform with B nanosheets as the carrier can capture negatively charged bacteria, and the NO generated after 808 nm illumination can better diffuse to the bacterial surface, thus achieving the ideal therapeutic effect of typical Gram-negative bacteria and Gram-positive bacteria (Lv et al., 2022) (Figure 5B). In conclusion, boron based photothermal nanomaterials still have a lot of space to explore in the field of antibacterial.

#### 3.2.2 Tellurium

Tellurium (Te) is a semiconductor metal, which belongs to the chalcogenide group together with Se and O. after the biological application of Se was developed, scientists were surprised to find that Te in the same group also has excellent biomedical application prospects. In fact, the antibacterial activity of Te was discovered earlier than the use of antibiotics (FLEMING and F. R. C. S., 1940), which also shows that tellurium is a promising antibacterial agent.

Lin et al. (2012) synthesized four different morphologies of Te NPs using TeO_2_ as raw material and N_2_H_4_ as reducing agent, and preliminarily explored their antibacterial activities, laying a foundation for the subsequent antibacterial application of tellurium nanoparticles. Tellurium is rarely natural, and most tellurium exists in the form of oxidized state, for example, −2 (telluride: Te^2−^), 0 (elemental element: Te^0^), + 4 (tellurite: TeO_3_^2−^), and + 6 (tellurite: TeO_4_^2−^) (Vahidi et al., 2021). The antibacterial effect of inorganic Te nanoparticles alone is not ideal (Lin et al., 2012). In order to expand its efficacy without affecting the performance of nanoparticles, many researchers choose to dope tellurium with other metals or some non-metallic materials or functionalize the synthesized tellurium nanoparticles to enhance the therapeutic effect. Brown D. et al. coated polyvinylpyrrolidone (PVP) on the surface of Te nanorods and analyzed the parameters representing the bacterial growth lag time *λ*, it was confirmed that the coating of PVP could delay the maturation of bacteria and then inhibit the growth of bacteria (Brown et al., 2018). However, the toxicity of tellurium nanoparticles remains a concern.

In order to reduce the toxicity of Te, scientists have made many attempts. On the one hand, plant extracts were selected as precursors for the synthesis of tellurium NPs (Zambonino et al., 2021). On the other hand, the reducing agent or capping agent needed in the synthesis process is changed. For example, Rosales-Conrado et al. synthesized spherical Te nanomaterials of different sizes using extracts from different kinds of tea leaves (Rosales-Conrado et al., 2021); Medina Cruz et al. Synthesized cubic Te NPs with lemon juice and lime juice respectively, and confirmed that when the concentration of nanoparticles was greater than 15 μg ml^−1^, they had a significant inhibitory effect on MDR *E*. *coli* and MRSA (Medina Cruz et al., 2019); Huang et al. green synthesized triangular star Te NPs (Huang et al., 2020) with common fructose as reductant; Vahidi et al. synthesized Te NPs (Vahidi et al., 2021) with strong antioxidant and bactericidal properties using fungi.

The research data show that the size of nanoparticles has a significant impact on their antibacterial activity. In fact, the smaller size nanoparticles will show higher antibacterial activity than the larger size nanoparticles (Pormohammad et al., 2021). The lethality of tellurium NPs to bacteria is mainly due to the specific binding of tellurium oxyanion and GSH in bacterial cells, which makes TeO_3_^2−^ be reduced to Te (0) and accumulated in cells. At the same time, ROS is generated in cells due to Fenton reaction, which activates the oxidative stress reaction in cells and finally kills bacteria (Huang et al., 2022) (Figures 5C,D). Secondly, Te NPs can also damage the integrity of bacterial biofilms, resulting in impaired function of cell membranes or cell walls. However, according to the previous research results, the antibacterial properties of many tellurium NPs synthesized at present are not ideal. Based on the great photothermal properties of tellurium NPs, the combination of tellurium NPs and PTT has gradually become a new popular trend. Wu et al. (2022) used cefotaxime (CTX)-loaded tellurium nanoparticles (Te-CTX NPs) as a photothermal agent, which, when irradiated by laser, produced a combined antibacterial effect of two synergistic pathways of PTT and CTX. On the one hand, Te restores the sensitivity of CTX to MRSA; on the other hand, the high temperature effect induced by PTT can destroy the antioxidant defense system of bacteria (Figure 5E). Under the synergistic effect of the two, 50 μg ml^−1^ Te-CTX NPs could effectively kill more than 90% of MRSA. The successful synergistic treatment of tellurium NPs and PTT provides more directions and possibilities for subsequent experiments. There are still more unknown about the combined use of the two.

### 3.3 Carbon-based nanomaterials

#### 3.3.1 Carbon quantum dots

Carbon quantum dots (CQDs) generally refer to carbon-based nanomaterials with a size less than 10 nm. The small size gives CQDs excellent photostability, water dispersibility, photocatalytic activity, biocompatibility, and bacteriostatic properties (Mintz et al., 2019). The excellent optical properties of CQDs have been widely used in the biomedical field in recent years (Nekoueian et al., 2019). In addition to being widely used in biological imaging, photodynamic therapy and photothermal therapy of tumors (Liu et al., 2022), it is also widely used in antibacterial.

As a promising photothermal inorganic nanomaterial, it has been reported that CQDs have intrinsic antibacterial properties in the blue light region of 400–470 nm. Under the excitation of 470 nm blue light, carbon dots can generate ROS by light to destroy MRSA and *E. coli* (Wang B. et al., 2021). The greatest advantage of CQDs is that they can be easily removed from the body and have low biological toxicity (Chung et al., 2020). The absorption coefficient of CQDs is low in the NIR region, but the temperature can be increased to the required range by adjusting the power density of the irradiated laser or doping other elements or modifying (Geng et al., 2020). Some researchers have synthesized composite fluorescent carbon dots (CsWO_3_-FCD) with the function of detecting and clearing bacteria. The carbon dots were coated with tungsten oxide (CsWO_3_). On the one hand, it can target bacteria; on the other hand, it can also use the strong NIR absorption of catechol and CsWO_3_ on the FCD surface to achieve photothermal antibacterial. At a small dose, it can effectively kill most *E. coli* and *S. aureus* within 5 min (Robby et al., 2021).

**FIGURE 6:**
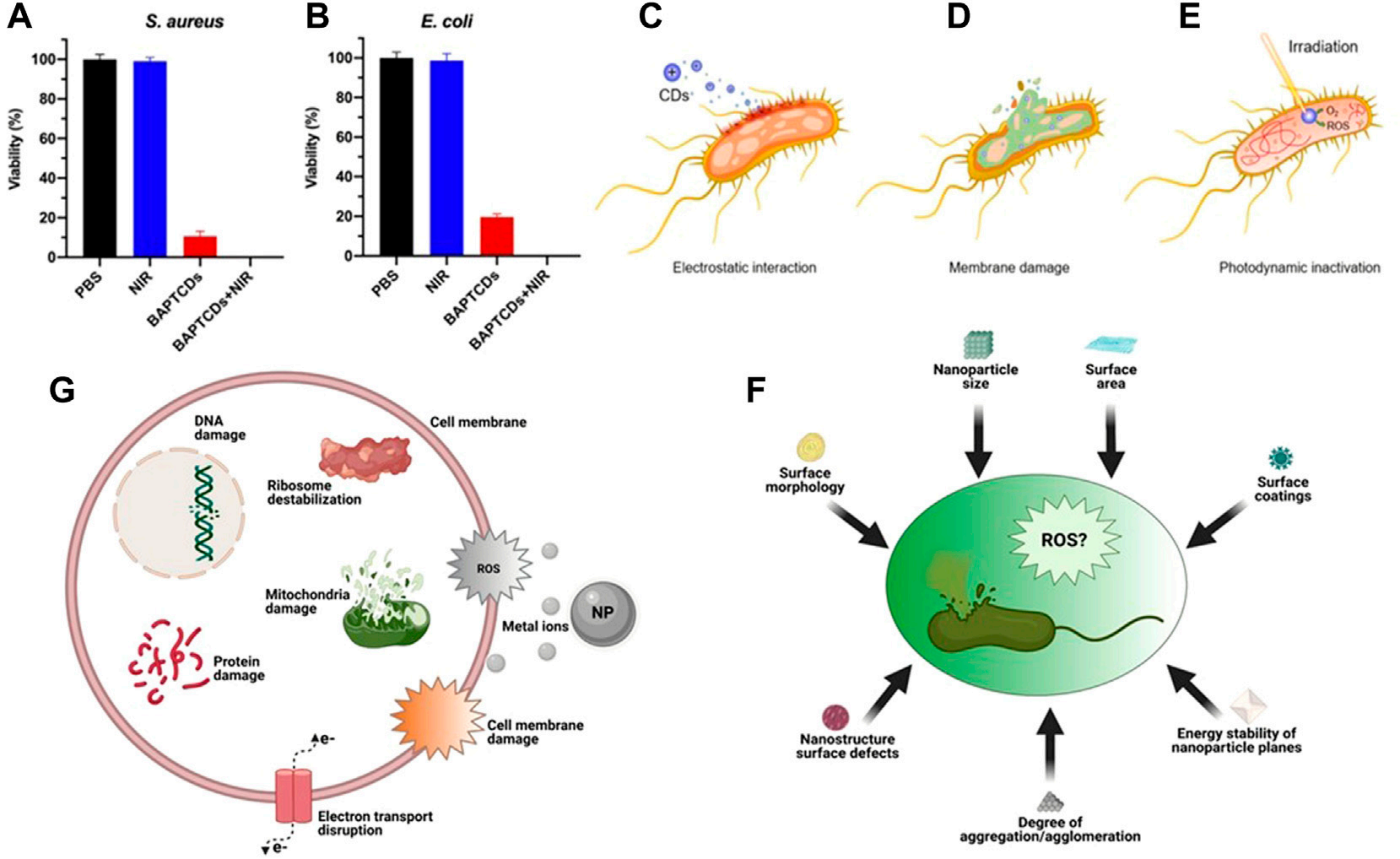
Bacterial viability of *E. coli* **(A)** and *S. aureus* **(B)** were obtained by the colony-forming count method. Reproduced from (Qie et al., 2022) with permission from Frontiers in Bioengineering and Biotechnology. General bactericidal mechanisms of action of CDs. **(C)** Electrostatic interactions between CDs and bacterial cell walls. **(D)** Internalization of CDs leads to irreversible damage caused by cytoplasmic leakage **(E)** CDs generate ROS under laser irradiation causing DNA damage. **(F)** Some factors of ROS production induced by carbon points and inhibition of bacteria. **(G)** Some mechanisms of toxicity of NPs. Reproduced from (Yougbare et al., 2021b) with permission from International Journal of Nanomedicine.

Qie et al. (2022) functionalized CQD with chiral biomolecules to prepare a bacterial affinity photothermal carbon point (BAPYCDs) for MurD ligase, and combined 808 nm 1.5 W cm^−2^ NIR laser with BAPYCDs. In 10 min, the BAPYCDs temperature of 200 μg ml^−1^ can be raised to 70.3°C. The temperature shows that BAPYCDs has excellent photothermal conversion efficiency. The addition of chiral molecule D-glutamic acid (D-Glu) enables BAPYCDs to be targeted and can be accurately localized to bacterial infection sites (Xin et al., 2017). When BAPYCDs were used alone for *in vitro* bacteriostatic experiments, 89.27% of *S. aureus* and 80.33% of *E. coli* were killed. When the above carbon dots were combined with NIR laser, almost all *S. aureus* and *E. coli* were killed (Figures 6A,B).

The antibacterial effect of the above CQDs is closely related to the charge and N content on the surface of CQDs (Verma et al., 2020). Most of the CQDs synthesized with amines or quaternary ammonium salts have certain antibacterial properties, but only the cationic groups of CQDs have limited antibacterial effect. Positively charged CQDs and negatively charged bacteria enter the cell interior through electrostatic interaction (Figure 6C) and cause cell wall damage and cytoplasmic leakage (Figure 6D) (Ghirardello et al., 2021), Irradiation with NIR raised the local temperature of bacteria and produced ROS, which further accelerated bacterial apoptosis (Figure 6E).

#### 3.3.2 Graphene and its derivatives

Since the discovery of graphene in 2004, graphene has become a hot scientific research field due to its good thermal conductivity and optical transmittance, high intrinsic mobility, large specific surface area and excellent conductivity (Yang et al., 2016). With the continuous expansion of the research field of photothermal nanomaterials, graphene and its derivatives have also become one of the research objects. The full-spectrum absorption properties of graphene and its derivatives make it very representative in the field of photothermal materials, which are widely used in antibacterial applications. The excellent heat transport characteristics also give graphene more important practical significance. When covid-19 broke out in 2019, in order to better resist the virus, Zhong et al. (2020) prepared a graphene coated mask with both photothermal and hydrophobic properties. Under the Sun, the coating can quickly heat up and kill bacteria. Wang H. et al. (2020) prepared a graphene quaternary ammonium salt platform functionalized by boric acid. The platform can not only use the targeting effect generated by electrostatic adsorption to mediate the antibacterial effect, but also synergistically enhance the antibacterial effect through the high temperature generated by 808 nm NIR laser irradiation. The experiments show that the nano platform can effectively treat biofilm infection and multi drug resistant gram-negative bacterial infection. In addition to the above-described PTT using laser light of the first near infrared window (NIR-I), PTT in the second near infrared window (NIR-II) has also been reported in recent studies. Geng et al. (2022) Selected 1,064 nm (NIR-II) NIR laser with stronger penetration ability than NIR-I as the treatment light source and graphene quantum dots (N-GQDs) doped with graphitized nitrogen as the treatment platform. Under the irradiation of 1,064 nm laser, N-GQDs showed nearly 100% inhibition and biofilm efficiency against multidrug-resistant bacteria. It also showed a function of promoting wound healing in a mouse model after being infected with MRSA.

Most of the graphene-based materials reported at present have relatively sharp edges and high photothermal conversion efficiency. Therefore, in addition to using photothermal effect to kill bacteria, mechanical/physical destruction can also be used to kill bacteria (Xin et al., 2019). The antibacterial mechanism is mainly to destroy the cell membrane and cell wall of bacteria.

To sum up, the antibacterial mechanism of the common photothermal conversion nanomaterials at present mainly includes three viewpoints: first, the generation of ROS (Figure 6F), and the damage mechanism of ROS to bacteria can be summarized into two ways: 1) Destroying the permeability of plasma membrane, causing some substances to flow out, or affecting the metabolic activities of bacteria (Ma et al., 2021); 2) DNA strand breaks and depolymerizes to produce stable oxidation products (Gao et al., 2017; Wentao et al., 2019); Second, release metal ions to destroy bacterial DNA and proteins (Figure 6G); Third, NPs gather on the surface of bacterial cell membrane and destroy the cell membrane and cell wall (Qi et al., 2020), blocking transmembrane electron transfer. The damage of cell membrane or cell wall will lead to the dysfunction of bacteria or the leakage of cytoplasmic components, and eventually cause the death of bacteria (Xin et al., 2019).

## 4 Summary and prospect

The toxicity and potential side effects of inorganic nanomaterials limit their application in the treatment of bacterial infection to a certain extent (Pormohammad et al., 2021). At present, modifying the synthesized inorganic nanoparticles or doping with other metal or non-metallic elements is an effective measure to deal with the toxicity of materials. However, in the specific implementation process, it often needs to face the problems of complex reaction process and harsh reaction conditions. The introduction of PTT successfully avoids the above-mentioned problems, and can achieve a more ideal bacteriostatic effect when using less nanomaterial dosage.

In recent years, the development of inorganic nanomaterials has also created more convenience for the use of PTT. More and more metal and non-metal inorganic nanomaterials with different sizes, morphologies and synthesis methods have shown excellent bacteriostatic effects in the process of combined use with PTT. However, some complications such as skin rash, allergic reaction, itching, etc. of these bactericides synthesized at present may still exist (Punjataewakupt et al., 2019). We still need to conduct a large number of clinical experiments to prove the safety of inorganic photothermal nanomaterials in terms of antibacterial.

We hope to turn the existing challenges into opportunities and develop inorganic photothermal nanomaterials that can be used for both antibacterial and wound healing in the near future.
