# Supplementary material for: Inorganic nanomaterials for intelligent photothermal antibacterial applications
Source: Front Bioeng Biotechnol. 2022 Oct 21;10:1047598. doi: 10.3389/fbioe.2022.1047598 (PMC9633683; doi:10.3389/fbioe.2022.1047598)
Supplement: Supplementary file 1 [file Table1.DOCX]

Table 1 Summary of inorganic nanomaterials for NIR photothermal antibacterial.

| Nanoparticles | Advantage | Treatment strategy | References |
| --- | --- | --- | --- |
| Au NPs | It has strong light absorption, surface plasmon resonance and photothermal effect. | When used in photothermal therapy, the lattice is heated, the temperature of the material rises, and bacteria are killed. | 51-61 |
| Ag NPs | It has adjustable surface plasmon resonance effect, has certain antibacterial properties and is easy to synthesize. | Ag itself can interact with phosphate and other substances inside and outside the cell, and inhibit the growth of bacteria. When combined with NIR, it can destroy the cell membrane and lead to bacterial death due to local heating. | 62-67 |
| B NPs | It has the functions of anti-inflammation and metabolic regulation, can maintain the stability of cell membrane and has targeting. | The existence of element B promotes the separation of holes and electrons, improves the optical properties, and can produce more ROS to kill bacteria under light conditions. | 68-76 |
| Te NPs | It can specifically bind to glutathione in cells and has a certain antibacterial effect. | Te (0) accumulates in cells, produces ROS under NIR laser irradiation and activates intracellular oxidative stress, resulting in the death of bacteria. | 77-88 |
| CQDs | It has small size, good water dispersibility, biocompatibility and optical properties, and is easy to be removed from the body. | CQDs can produce ROS under laser irradiation, and the temperature increases at the same time. | 89-99 |
| Graphene | It has good thermal conductivity and optical transmittance and has full spectrum absorption. | Graphene itself has a targeted effect on bacteria, graphene produces high temperature under NIR laser irradiation, and the two synergistically enhance the antibacterial effect. | 100-109 |
